# Redox Regulation in Diazotrophic Bacteria in Interaction with Plants

**DOI:** 10.3390/antiox10060880

**Published:** 2021-05-30

**Authors:** Karine Mandon, Fanny Nazaret, Davoud Farajzadeh, Geneviève Alloing, Pierre Frendo

**Affiliations:** 1 Université Côte d’Azur, INRAE, CNRS, ISA, 06903 Sophia Antipolis, France; Karine.MANDON@univ-cotedazur.fr (K.M.); fanny.nazaret@inrae.fr (F.N.); alloing@unice.fr (G.A.); 2 Department of Biology, Faculty of Basic Sciences, Azarbaijan Shahid Madani University, Tabriz 5375171379, Iran; farajzadeh@azaruniv.ac.ir; 3 Center for International Scientific Studies and Collaboration (CISSC), Ministry of Science, Research and Technology, Tehran 158757788, Iran

**Keywords:** bacteria, diazotrophs, plant symbiosis, redox homeostasis, ROS

## Abstract

Plants interact with a large number of microorganisms that greatly influence their growth and health. Among the beneficial microorganisms, rhizosphere bacteria known as Plant Growth Promoting Bacteria increase plant fitness by producing compounds such as phytohormones or by carrying out symbioses that enhance nutrient acquisition. Nitrogen-fixing bacteria, either as endophytes or as endosymbionts, specifically improve the growth and development of plants by supplying them with nitrogen, a key macro-element. Survival and proliferation of these bacteria require their adaptation to the rhizosphere and host plant, which are particular ecological environments. This adaptation highly depends on bacteria response to the Reactive Oxygen Species (ROS), associated to abiotic stresses or produced by host plants, which determine the outcome of the plant-bacteria interaction. This paper reviews the different antioxidant defense mechanisms identified in diazotrophic bacteria, focusing on their involvement in coping with the changing conditions encountered during interaction with plant partners.

## 1. Introduction

**Figure 1:**
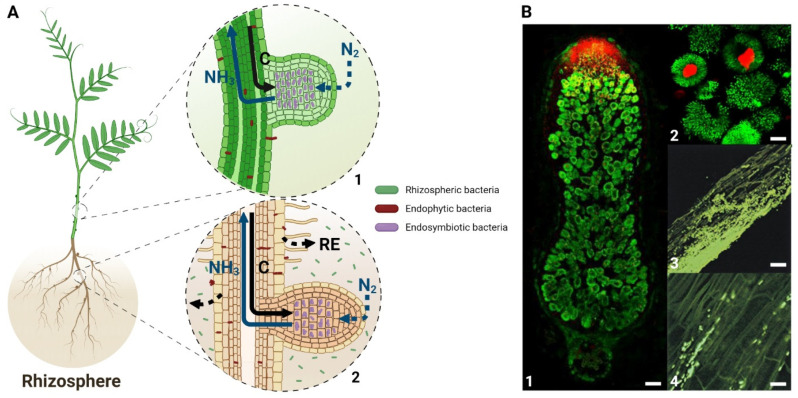
Colonization patterns of endosymbiotic and endophytic bacteria in interaction with the plant. (**A**) Schematic representation of bacterial distribution within the plant. Rhizospheric bacteria (green) spread into the rhizosphere but do not directly interact with plant tissues. Endophytic bacteria (red) colonize the plant surface and intercellular space of plant tissues. Endosymbiotic bacteria (purple) are able to induce the formation of the stem (**A1**) or root (**A2**) nodules containing nitrogen-fixing bacteroids. In exchange for the ammonia provided by these endosymbiotic bacteria, plant transfer carbohydrates derived from photosynthesis. N_2_: atmospheric nitrogen; NH_3_: ammonia; C: carbohydrates; RE: root exudates. (**B**) Pictures of endosymbiotic and endophytic bacterial distribution within plants. For endosymbiotic bacteria, Live/Dead microscopic analysis of mature nodules (**B1**) and infected cells in zone III (**B2**) formed during *M. truncatula*/*S. meliloti* symbiosis is represented. For endophytic bacteria, longitudinal sections of a maize root colonized by GFP-labelled *Klebsiella pneumoniae* (**B3**; **B4**) are represented reprint from reference [9] with kind permission from the American Society for Microbiology. Scale bar: 100 µm (**B1**), 10 µm (**B2**; **B4**), or 20 µm (**B3**).

Diazotrophic rhizobacteria that interact positively with plants involve endophytic bacteria and rhizobial endocellular symbionts. Their benefit on plant growth has been attributed to a variety of single or combined mechanisms, including phosphate solubilization, siderophore synthesis, production, and secretion of several phytohormones as well as biological nitrogen (N_2_) fixation (BNF), leading to enhanced plant nutrition [1]. Nitrogen (N) is one of the major macronutrients for plants, which plays a fundamental role in protein synthesis and energy metabolism through photosynthesis, and its deficiency affects plant development [2]. The addition of chemical fertilizers is a major source of N for crops, which not only requires fossil fuels for their production but increases water pollution as well. Contributing to more than half of total crop N demand, BNF is a possible alternative to fertilizers [3]. The BNF is exclusively performed by prokaryotes, archaea, and bacteria, collectively known as diazotrophs. For archaea, BNF is performed by diazotrophic methanogens, which produce methane [4]. Diazotrophic bacteria encode a specific protein complex, the nitrogenase that reduces the atmospheric N_2_ to ammonia (NH_3_). The diazotrophs such as *Azotobacter* sp., *Azospirillum* sp., *Bacillus* sp., *Cyanobacteria* sp., and *Clostridum* sp. are free-living bacteria present in the rhizosphere, which can form endophytic interactions with a wide variety of plants (see Figure 1) [5]. In parallel, endosymbiotic diazotrophs reside intracellularly in living plant cells and induce the formation of differentiated plant structures as in the case of *Nostoc* sp. with the *Gunneraceae* family [6], *Frankia* sp. with the actinorhizal plants [7], and the *Rhizobia* sp. with the *Fabaceae* family [8]. In the two latter cases, the bacteria are hosted in the stem or root nodules that are the neoformed plant organs involved in BNF.

In plants and microbes interactions, the plant immune system is differentially modulated by the microorganisms, depending on the type of interaction [10,11]. Upon infection, plant receptors recognize a wide range of conserved bacterial molecules, referred to as microbe-associated molecular patterns (MAMPS) allowing the induction of plant defense mechanisms [12]. As part of the defense responses that follow MAMP receptor activation, oxidative and nitrosative bursts occur, both to control microorganism colonization and to spread a systemic defense signal throughout the plant [13]. The interacting bacteria have evolved systems to suppress plant defense reactions; they concomitantly have set up systems to cope with plant antimicrobial compounds, in particular non-enzymatic antioxidant molecules and Reactive Oxygen Species (ROS)/Reactive Nitrogen Species (RNS) detoxification enzymes (review in [14]).

Whereas oxidative burst during plant-pathogen interactions has been extensively described, studies concerning the modulation of redox activities in plants interacting with beneficial microbes remain sparse. However, modulation of antioxidant enzyme activities has been observed during some interactions between plants and plant growth-promoting rhizobacteria (PGPR) [15,16]. Analysis of sugarcane root transcriptome showed that many genes involved in the redox process were upregulated and that peroxidase and catalase enzyme activities were also significantly upregulated in response to the *Burkholderia anthina* MYSP113 [16]. Similarly, up-regulation of genes encoding ROS scavenging enzymes was observed in Arabidopsis, rice, and tomato roots inoculated with *Azospirillum brasilense* [17,18]; peroxidase accumulation was also observed in rice roots inoculated with *Sinorhizobium meliloti* [19]. In contrast, a decrease in the expression of *superoxide dismutase*, *catalase* and *ascorbate peroxidase* genes is detected in maize roots inoculated with *A. brasilense* [20], and inoculation of wheat with *A. brasilense* leads to limited production of O_2_^•−^ from the root plant [21]. Altogether, these data suggest a complex pattern of adaptation during plant colonization as observed for the rhizobial infection of legumes.

Besides the endophytic interactions, plant-rhizobia interactions involving an intracellular symbiosis have been more deeply characterized. The mutualistic interaction of rhizobia with legume plants involves two major biological processes. The first one is the entry of the bacteria into the plant, followed by the bacterial transfer to the nodule primordia, then entering into plant cells in the nodule infection zone. In compatible interactions, the nodulation (Nod) factors excreted by the bacterial partner are recognized by the host plant receptors and elicit host responses such as root hair curling and invasion by the bacteria. During these early steps, there is production of ROS involved in the specific recognition of the bacteria [22,23]. The production of ROS has been detected a few minutes after Nod factor treatment [24]. The transfer to the inner cortex cells is performed through the infection thread (IT), a transcellular apoplastic compartment, which grows in parallel with the bacterial cell division [25]. Inhibition of ROS production impairs the root hair curling and the IT formation [26]. Respiratory Burst Oxidase Homolog Gene (RBOH) A is crucial for rhizobium infection in the common bean, suggesting that NADPH oxidases are responsible for the ROS production in the symbiotic interactions [27]. ROS production is also involved in the oxidative cross-linking of the IT matrix that allows an efficient transfer of the dividing bacteria from the plant epidermis to the inner cortex [28,29]. In addition to this general infection model, some rhizobia enter the plant through a mechanism called crack entry, which is a characteristic feature of some subtropical legumes belonging to the dalbergoid/genistoid clades [30]. This infection process involves the formation of infection pockets that was shown to be associated with localized plant cell death and the production of large amounts of ROS [31].

The second process is the differentiation of plant and bacterial cells, which occurs in the infected cells of the nodule to allow the setup of BNF. In the plant cell, bacteria are surrounded by the symbiosome membrane, which derives from the infected cell plasma membrane. At this time point, bacteria undergo a differentiation process allowing the formation of N-fixing bacteroids that release ammonia into the host cell in exchange for reduced carbon and other nutrients from the plant. Some leguminous plants, such as the ones from the inverted repeat-lacking clade, including *Medicago truncatula*, induce terminal differentiation of their symbionts, which prevents their division before they fix nitrogen. In this plant model, the production of hydrogen peroxide (H_2_O_2_) has been observed in the infection zone [32]. Besides the production of ROS in the infection zone, modification of the bacterial redox balance is also associated with the production of Nodule Cysteine Rich (NCR) peptides that are specifically expressed by the plant partners in the nodule infection and nitrogen-fixing zones [8,33]. The biological activity of these peptides is regulated by their redox state [34,35], and specific plant thioredoxins targeted to the bacteroids are able to modulate their redox state and the bacteroid differentiation [36].

Once differentiated, the bacteroid reduces N_2_ to NH_3_ in the nodule nitrogen-fixing zone (NFZ), where microoxic conditions prevail to allow nitrogenase functioning. These conditions involve the presence of a structural barrier in the nodule cortex and the expression of leghemoglobins in the NFZ [37]. The lower level of oxygen does not abolish the functioning of NADPH Oxidase in this NFZ. In *M. truncatula*, *MtRBOHA* is specifically expressed in the NFZ, and its activity was involved in nitrogen fixation efficiency [38]. Recently, four RBOHs from soybean (*Glycine max*) showed strong expression in nodules, pointing to their probable involvement in the nodulation process [39]. The importance of redox regulation in the plant symbiotic partner is also noticeable by the significantly higher level of antioxidant molecules in nodules than in roots [40]. Modification of the glutathione content in the plant partner affects both the nodule formation and functioning [41,42] showing that the plant antioxidant defense is crucial for the setup and the functioning of the nitrogen-fixing symbiosis. Similarly, methionine sulfoxide reductase B has been shown to play an essential role in the nodule development and functioning in the symbiotic interaction between *Astragalus sinicus* and *Mesorhizobium* [43].

To cope with the redox modifications that occur during endophytic and endosymbiotic interactions with plants, bacteria need to adapt to their redox homeostasis. The review presents some general aspects of endogenous antioxidant systems in bacteria and then summarizes the recent knowledge concerning redox regulation mechanisms in diazotrophic bacteria during beneficial plant-bacterial interactions.

## 2. The Bacterial Response to ROS

Aerobic bacteria have to cope with endogenous ROS derived from their metabolism. Exposure to abiotic stresses such as heat, low pH or salt, can also induce the formation of intracellular ROS [44]. In addition to these endogenous sources of redox stress, bacteria interacting with eukaryotic hosts are exposed to ROS produced by the hosts. These molecules are deleterious to biological macromolecules, including proteins, lipids, and DNA, and their concentration must be tightly controlled [45]. When present at the proper concentrations, ROS can function as signal molecules and activate multiple signal transduction pathways within the bacterial cells [46,47]. To maintain their intracellular redox homeostasis, bacteria use enzymatic and non-enzymatic defense systems. Both constitutive and inducible mechanisms are triggered by bacteria to manage the cellular redox environment and adapt to different lifestyles.

**Figure 2:**
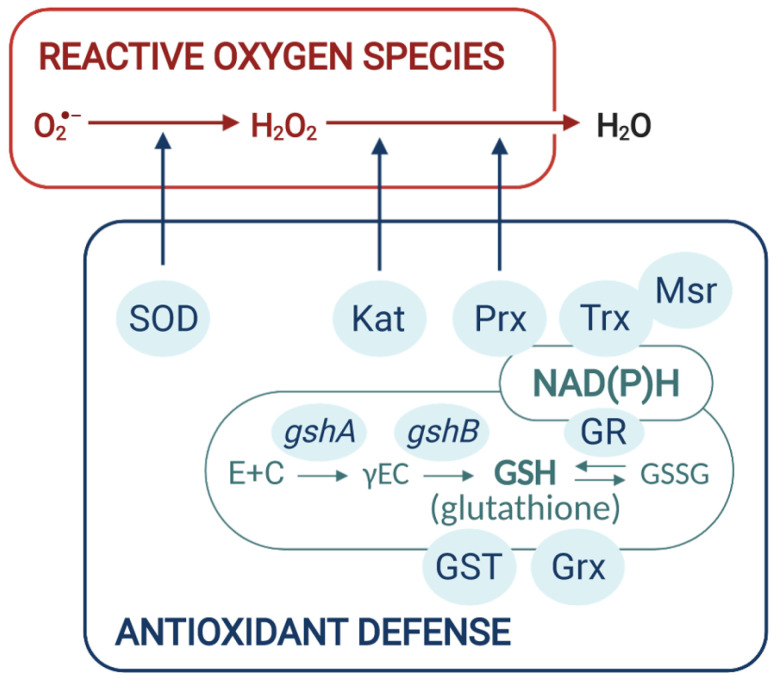
General bacterial antioxidant defense. Bacteria protect themselves from ROS thanks to enzymes (SOD: superoxide dismutase; Kat: catalase; Px: peroxidase; Prx: peroxiredoxin; Trx: thioredoxin; Msr: methionine sulfoxide reductase) and low molecular weight thiol glutathione (GSH). GSH can be used by glutathione-S-transferase (GST) or glutaredoxin (Grx) to detoxify ROS. Glutathione reductase (GR) allows the regeneration of GSH from the oxidized form GSSG. Grx works with Trx in order to reduce protein disulfides, and Msr reduces methionine sulfoxide residues to methionine. GR, Trx, and Msr use NAD(P)H as an electron donor.

Bacteria protect themselves from ROS by producing various scavenging enzymes. For instance, the metalloenzyme superoxide dismutase (SOD) converts O_2_^•−^ to H_2_O_2_, which is reduced to H_2_O by enzymes such as monofunctional catalases, bifunctional catalase-peroxidase (Kat), or thiol peroxidases such as peroxiredoxins (Prx) (Figure 2). The peroxiredoxin family has been divided into two categories, the organic hydroperoxide resistance protein Ohr, which displays a high affinity for long-chain fatty acid peroxides, and the TSA/AhpC subgroup containing 1-cys, 2-cys peroxiredoxins, and the AhpCD system that have a broad range of substrate including H_2_O_2_, organic and fatty acid peroxides. ROS can also oxidize methionine residues of proteins to methionine sulfoxide, thus altering protein structure. The dedicated methionine sulfoxide reductases (Msr) enzymes reduce methionine sulfoxide residues back to methionine [48].

Low molecular weight thiol-redox buffers also function in the detoxification of ROS. Tripeptide glutathione (γ-glutamyl-cysteinyl-glycine, GSH), the major thiol reductant in eukaryotes and many bacteria, participates in the intracellular defense against ROS-induced oxidative damage and maintains the intracellular compartment in a reduced state. GSH can remove free radicals, e.g., superoxide anion, hydroxyl radical, nitric oxide, and carbon radicals via direct interaction. It is also a cofactor for various antioxidant enzymes such as glutaredoxins (Grx) and glutathione-S-transferase (GST) to detoxify ROS, xenobiotics and/or heavy metals [49,50]. The resulting oxidized form of glutathione (GSSG) is reduced to GSH by the NAPDH-using enzyme glutathione reductase (GR). GSH is synthesized in a two steps procedure. The first step, in which glutamate is ligated with cysteine to form γ-glutamylcysteine, is catalyzed by the γ-glutamylcysteine synthetase (γ-ECS) encoded by *gshA*. GSH synthesis is then completed by the addition of glycine to γ-glutamylcysteine, which is mediated by the glutathione synthetase (GSHS) encoded by *gshB*. Under oxidative-stress conditions, GSH can form reversible mixed disulfides with reactive protein thiols, protecting them against over-oxidation. This S-glutathionylation can serve to transduce a redox signal by modulating protein function [51]. S-deglutathionylation, that is, the reduction of GSH-protein mixed disulfides is catalyzed by Grxs in a coupled system with GR and NADPH as an electron donor. Grxs together with Trxs are also involved in the reduction of protein disulfides. The oxidized Trx is reduced by thioredoxin reductase, receiving electrons from NADPH (Figure 2).

## 3. Redox Regulation in Rhizosphere and Root Colonization

Endophytic and endosymbiotic bacteria live in the rhizosphere in close association with the root surface, where plant-derived compounds such as amino acids or carbohydrates favor the proliferation of heterotrophic aerobic bacteria. It is also a place where bacteria have to deal with abiotic stresses such as osmotic and pH alterations or the presence of toxic compounds. Some root exudates are specific to particular plant species, like original carbon and nitrogen sources or secondary metabolic compounds such as flavonoids [52]. Despite their capacity to attenuate the induction of the plant defense, endophytic and endosymbiotic bacteria have to face oxidative stress during root adhesion and penetration. Adaptation to the rhizosphere is thus critical for the efficiency of root colonization and the outcome of plant-bacteria interactions.

*Azospirillum* sp. is a well-characterized PGPB due to its capacity to colonize the roots of major cereal crops and grasses. Different genetic determinants have been proposed to contribute to the large host range of *Azospirillum* sp. and may account for the niche-specific adaptation of the bacteria to a given rhizosphere. In particular, genes related to ROS detoxification and multidrug efflux seem to be involved in *A. lipoferum* 4B adaptation to the rice rhizosphere [53,54]. A comparative analysis of *Azospirillum* sp. genomes shows that several enzymes involved in the oxidative stress response differ among *Azospirillum* species [55]. As an example, only one gene encoding a monofunctional catalase was identified in *Azospirillum* sp. B510, while two genes, one encoding catalase and the other encoding a bifunctional catalase peroxidase, were encountered in *A. lipoferum* 4B. A comparative genome-wide transcriptome analysis performed with the *A. lipoferum* strain 4B following inoculation on original host and non-natural host rice cultivars, highlights common and cultivar-dependent transcriptomic responses [53]. During interaction with both cultivars, genes involved in redox homeostasis encoding an Ohr-like protein, a Grx, and a GST, were upregulated. Similarly, the expression of genes encoding methionine sulfoxide reductases (*msrA* and *msrB*) and a 2Fe-2S ferredoxin-like protein was also enhanced. By contrast, a specific induction of thioredoxin-encoding genes (*trxB* and *trxC*) and *sod*-like gene was observed upon inoculation of the non-host cultivar. The latter result supports the idea that bacteria interacting with a less adapted host perceive stronger oxidative stress suggesting a better adaptation of *A. lipoferum* 4B towards its original host cultivar. RNA-seq analysis of *A. brasilense* FP2 on wheat revealed induction of a *sod*-like gene (*sodB*), suggesting the activation of the antioxidant defense during the interaction with plants [56]. In addition, the *A. brasilense* Sp245 genome contains an *ahpC* gene that was shown to be involved in resistance towards H_2_O_2_ and organic alkyl hydroperoxide stress [57]. However, the *ahpC* inactivated mutant is not impaired in the colonization of wheat roots, even in competitive experiments with the wild-type strain. The multiplicity of ROS scavenging enzymes in the bacteria might be the reason for the absence of phenotype. Indeed *A. brasilense* sp7 contains three catalase-encoding genes, *katAI*, *katAII*, and *katN* [58].

“Omics” analyses were also performed in endophytes other than *Azospirillum* sp, and results concerning the importance of the antioxidant defense of the studied bacterial strains are not always conclusive. The genome of *Leifsonia* sp. Ku-ls isolated from rice presents an elaborate antioxidant defense [59]. A proteome analysis has been performed on *Herbaspirillum seropedica*, a bacterium able to colonize roots of gramineous plants of economic importance, such as wheat, maize, rice, or sugarcane (review in [5]). As protein secretion plays a fundamental role in the adaptation of bacteria to its environment and during plant-bacteria interaction, the secretome of *H. seropedicae* was performed using 2D electrophoresis [60]. Among the 41 proteins, the most important secreted protein was an NADPH:quinone oxidoreductase, and to a lesser extent, a peroxiredoxin and a Fe-superoxide dismutase (SOD). However, the secretome was extracted from bacteria in laboratory culture conditions and the presence of Fe-SOD during plant-bacteria interaction has not been investigated. In *Gluconoacetobacter diazotrophicus,* ROS level is increased in nitrogen-fixing cells, where a concomitant induction of genes related to the antioxidant defense, *sodA*, *katE*, *kat*, *katC*, and genes involved in glutathione cycle was emphasized [61]. Following inoculation on rice, the expression of genes encoding catalases, SOD, and GR was shown to be induced from 30 min to 4 days post-inoculation, and the bacterial mutants *sod* and *gr* were poorly able to colonize rice (Table 1) [62].

Several transcriptomic approaches have also been performed to identify genes of endosymbiotic bacteria potentially involved in adaptation to the rhizosphere. Gene induction pattern in the rhizosphere or response to root secretions was compared against laboratory culture conditions, and numerous genes of the antioxidant defense in various rhizobia were thereby found to be upregulated. An efficient antioxidant system may be a prerequisite for symbiotic bacteria to deal with ROS in the rhizosphere and root surfaces, providing the required physiological adaptation to thrive in the plant root system. In one of these studies, the early adaptation of *Rhizobium leguminosarum* to the rhizospheres of pea (host-legume), alfalfa (non-host legume), and sugar beet (non-legume) was investigated in parallel [85]. This approach allows identifying genes specifically induced in the pea rhizosphere (138 genes), whereas others were found to be upregulated in all rhizospheres (106 genes). Some genes involved in response to oxidative stress belong to this common core of rhizosphere-induced genes, such as genes encoding MsrA and MsrB, a thioredoxin and a peroxiredoxin. Another example is a comparative analysis of the transcriptomes of three Mimosa symbionts, the α–proteobacterium *Rhizobium mesoamericanum* and the two β-proteobacteria *Bukholderia phymatum*, an ancient symbiont, and *Cupriavidus taiwanensis*, which has probably acquired symbiotic genes from a *Burkholderia* symbiont more recently. The upregulation of genes encoding putative peroxiredoxin, thioredoxin, and GST was shown to be part of the common response of the two β-proteobacteria to *Mimosa* root exudates, highlighting the importance of antioxidant defense in bacterial survival in the rhizosphere [86]. In addition to transcriptomic approaches, genome-wide approaches based on STM (signature-tagged mutagenesis) mutants were performed in *S. meliloti* and *R. leguminosarum* to identify genes important for rhizosphere colonization [72,87]. They showed that competitiveness in the rhizosphere is largely determined by metabolic functions and motility and by tolerance to abiotic stresses generated by root exudates as well [87]. Of the 170 *R. leguminosarum* genes involved in pea rhizosphere competitiveness, *sodB* is specifically involved in survival in the rhizosphere, a Grx-encoding gene is necessary for both rhizosphere bacterial growth and root colonization, while *gshA* and *gshB* are required for rhizosphere competitiveness and onward stages of symbiosis as well [72].

## 4. Antioxidant Defense in Legume Symbionts

### 4.1. ROS Scavenging Enzymes

A complex pattern of ROS production is required for root colonization by rhizobia and nodule development (reviewed in [88,89]). Accumulation of ROS during plant colonization, nodule development, and functioning has been highlighted in *Sesbania rostrata*, *Phaseolus vulgaris* and *M. truncatula* [31,90,91,92]. The microsymbiont also participates in the fine-tuning of ROS concentration during symbiosis. For example, using a pharmacological approach, *Bradyrhizobium* sp. peroxidase activity has been shown to be necessary for the early events of the interaction with *Arachis hypogaea* [93], while a *S. meliloti* strain that degrades H_2_O_2_ very efficiently is affected in plant colonization [28].

Many rhizobia contain multiple ROS detoxifying enzymes that are required for symbiosis (Table 1). The role of rhizobial antioxidant defense has been first characterized in *S. meliloti*. This bacterium possesses two SODs, SodA and SodM, three heme b-containing catalases in addition to one heme-chloroperoxidase [64,94,95]. The SodM protein is expressed in nitrogen-fixing bacteroids, and mutants inactivated in *sodA* displayed different symbiotic phenotypes depending on the genotype of the strain used. A periplasmic SodA protein was characterized in *Rhizobium leguminosarum bv. viciae* [96] and a SodA purified from bacteroids appeared the most abundant SOD isoenzyme in pea nodules [97]. In a transcriptomic analysis of *B. japonicum* exposed to H_2_O_2_, three distinct *sod* genes, *sodF*, *sodM* and a Fe/Mn-SOD-encoding gene were upregulated [98]; however, their roles *in planta* have not been established. In *S. meliloti*, the three catalase genes, *katA*, *katB*, and *katC*, are differentially expressed in culture and symbiosis. The H_2_O_2_ inducible *katA* gene is expressed in the nitrogen-fixing zone, while the stress-inducible *katC* is strongly transcribed in ITs [65]. The disruption of both *katB* and *katC* genes leads to a drastic phenotype, with aborted bacteroid differentiation following plant cell release. Besides, the double mutant *katAC* is able to differentiate into bacteroids that undergo premature senescence. In parallel, the overexpression of the bifunctional catalase-peroxidase KatB, thereby acting as a sink for H_2_O_2_ inside the ITs, results in a delayed nodulation phenotype associated with aberrant infection threads [28]. Catalases have been shown to be essential during symbiosis in rhizobia, such as *Mezorhizobium loti*, which has a monofunctional catalase KatE and a bifunctional KatG [66]. KatG is required in a free-living state for H_2_O_2_ scavenging during the exponential growth phase or H_2_O_2_ exposure, while KatE acting during the stationary phase is required to sustain optimal nitrogenase activity in symbiosis with *Lotus japonicus*. Peroxiredoxins act in concert with the catalases for degrading H_2_O_2_. *S. meliloti* contains an Ohr protein induced in presence of alkyl peroxide, not essential for symbiosis, and an H_2_O_2_-inducible AhpC-like protein present in bacteroid extracts [99,100,101]. In addition to the H_2_O_2_-inducible catalase KatG, *B. japonicum* contains multiple genes involved in redox homeostasis whose products are present in bacteroid extracts, two SOD, one catalase, the AhpCD system, and two non-heme chloroperoxidases [102,103]. In *Mezorhizobium huakii/A. sinikus* interaction, the peroxiredoxin PrxA is important for symbiosis as a *prxA* mutant elicits spherical nodules displaying a considerable reduction of nitrogen fixation activity associated with bacteria that fail to differentiate [68]. Besides, *R. etli* contains both a catalase KatG and a 2-cys peroxiredoxin PrxS, whose inactivation gives no phenotype [67,104]. However, disruption of both *katG* and the *prxS* results in a reduction of 40% in nitrogen fixation, suggesting that a compensation mechanism may occur between multiple antioxidant overlapping enzymes [67]. *Azorhizobium caulinodans* is unique among rhizobia, as the bacterium is both able to assimilate N_2_ in a free-living state and to induce the formation of stem and root nodules on the tropical legume *S.rostrata. A. caulinodans* possesses the catalase-peroxidase KatG and the alkylhydroperoxide reductase AhpCD to cope with H_2_O_2_ increase, which are differentially used depending on the growth phase and H_2_O_2_ concentration [69,70]. In addition, protection against organic hydroperoxide stress requires the Ohr protein [71]. The *katG*, *ahpC*, and *ohr* single mutants displayed a significant reduction in stem nodule number associated with a reduced nitrogen fixation ability. In contrast, the *S. rostrata* root nodules were not affected by the *katG* or the *ahpC* inactivation, suggesting that a specific adaptation of *A. caulinodans* is required in stem nodules to face the high O_2_ and derivatives produced by the photosynthetic cortical cells close to the infected cells. Lastly, hydroperoxidase detoxifying enzymes also appear to play a significant role in actinorhizal symbiosis. Indeed, a transcriptomic analysis of *Frankia alni* during symbiosis with *Alnus glutinosa* showed that genes encoding a Prx and a SOD were upregulated in nodules as compared to free-living conditions, while the *katA* gene was highly expressed in both conditions [105].

### 4.2. The Role of Bacterial GSH/Grx System

The GSH biosynthetic pathway plays a crucial role in rhizobia during both free-living and plant-associated lifestyles. GSH was first shown to be essential in *S. meliloti*, from which a *gshA* mutant could not grow in a minimal medium. Conversely, a *gshB* mutant was able to divide, even at a lower rate than WT, suggesting that γ-EC could partially compensate for GSH defect [74]. A growth defect in *gshB* mutants was also observed in other rhizobia [73,76,77]. GSH deficiency was linked to metabolic changes in *R. etli*, reducing glutamine uptake through the activity of Aap and Bra transporters [76]; in *R. leguminosarum,* a *gshB* mutant is affected in the uptake of several carbon source compounds such as glucose, succinate, glutamine, and histidine [73]. In addition, *gshB* inactivation causes constitutive oxidative stress associated with a higher catalase activity in *S. meliloti* and an increased sensitivity to peroxides in *M. huakuii* [74,78].The lack of bacterial GSH compromises the early steps of interaction. Specifically, *gshB* mutants have a lower capacity to colonize the host plant in the interaction between *R. leguminosarum* and *P. sativum* and are impaired in nodulation during *S. meliloti*/ *Medicago* sp., *R. etli*/*P. vulgaris*, *R. tropici/P. vulgaris* and *M. huakuii*/*A. sinicus* interactions. Furthermore, GSH has been shown to be crucial in nodule functioning. In most cases, *gshB* mutants produced abnormal nodules with a strong nitrogen-fixation deficiency, correlated with the early senescence of nodules and bacteroids [74,75,76,77,78]. In line with these findings, the recycling of GSH by glutathione reductase was also shown to be involved in nodulation and in nitrogen fixation ability [79]. In *R. tropici/P. vulgaris* interaction, GSH deficiency was also associated with increased production of superoxide anion [77]. Regarding the interaction between *S. meliloti* and *M. truncatula,* a combination of physiological, and molecular markers, microscopic and flow cytometry analyses, showed that a *gshB* mutant undergoes complete differentiation before being engaged in an early senescence process [75]. Totally, these results underline the importance of the bacterial GSH/GSSG redox couple for adaptation to the host-plant environment and survival inside the host cells (Table 1).

The contribution of GSH-dependent redox enzymes to symbiosis efficiency was highlighted by studies on different Grxs (Table 1). Grxs are divided into two classes based on the active site motifs: dithiol Grxs (class I) contain a typical CxxC motif, while monothiol Grxs (class II) possess a CxxS motif. Grxs of the two classes are present in different rhizobia, and their particular role has been investigated in *S. meliloti* and *A. caulinodans* during free-living and symbiotic lifestyles [80,81]. In *S. meliloti* on one side, dithiol Grx1 has been shown to contribute to the deglutathionylation of proteins. A *grx1* mutant has an intense growth defect and an increased sensitivity to H_2_O_2_ in bacterial cultures. During the interaction with *M. truncatula,* the *grx1* mutant induces abortive nodules with infected cells containing no differentiated bacteroids. On the other side, the monothiol Grx2 of *S. meliloti* is involved in Fe metabolism. A *grx2* mutant compared with the WT strain grows more slowly and contains an increased concentration of free Fe and decreased activity of Fe-S cluster containing enzymes. Inactivation of *grx2* affects nodulation with *M. truncatula* and the nitrogen-fixing capacity of bacteroids. Those bacteroids are fully differentiated, which suggests that the mutation has a direct effect on the Fe-S cluster of nitrogenase. Likewise, mutants in the *suf* operon, involved in Fe/S cluster formation, also have a lowered nitrogen fixation capacity in *S. meliloti* and *A. caulinodans.* Furthermore, the *suf* operon of *F. alni* is one of the most up-regulated clusters in nodule bacteria compared to free-living cells [105,106,107].

The genome of *A. caulinodans* contains two Grxs of the class II (Grx1 and Grx2) and two Grxs of the class I (Grx3 and Grx4). Using a combination of single, double, and quadruple mutants, Cao and colleagues showed that genes encoding either monothiol or dithiol Grxs exhibit functional redundancies under normal growth conditions and in the presence of H_2_O_2_ and organic hydroperoxides [81]. The specific inactivation of the two monothiol Grxs results in lower GSH/GSSG ratio and Fe content, suggesting that they participate in Fe and redox homeostasis. In the symbiosis with *S. rostrata*, *grx1 grx2* and *grx3 grx4* double mutants have a reduced nitrogen fixation capacity, and the quadruple mutant, even more, showing a collective contribution by the two Grxs classes to the N_2_ fixation efficiency.

## 5. Redox-Based Transcriptional Regulation in Endophytic and Endosymbiotic Bacteria

ROS can cause reversible post-translational modifications and induce conformational changes of proteins, often by targeting thiol groups of redox-active cysteines. This mechanism contributes to redox signaling, notably by triggering activation or inactivation of redox-sensing transcriptional regulators (TR) [108]. In general, TRs are essential for bacteria to rapidly respond to environmental changes, improving their adaptation plasticity, cellular homeostasis, and colonization of new niches. Thiol-based redox regulators particularly play an essential role in the control of oxidative stress response and adaptation to the host-environment. Several have been characterized in rhizobacteria, and their role in symbiotic interaction was underlined in some cases.

OxyR is a redox-sensitive regulator of the LysR family, which is conserved in Gram+ and Gram- bacteria and regulates the response to oxidative stress. OxyR binds as a tetramer to promoter regions of target genes and acts as an activator or a repressor depending on whether it is oxidized or reduced. In many bacteria, OxyR regulates the expression of genes involved in peroxide detoxification pathways, protein repair, and other oxidative defense-related genes. OxyR in *S. meliloti* was initially shown to contribute to H_2_O_2_ adaptation and both represses and activates the expression of *katA* depending on its oxidation state [109]. The OxyR regulon has been determined in a whole-genome transcriptional analysis of WT strain and *oxyR* mutant exposed to H_2_O_2_ [101]. It contains over 100 genes, of which some encoding antioxidant functions such as the already known KatA, an AhpC-type alkyl hydroperoxidase, and two chloroperoxidases. *OxyR* is overexpressed during symbiosis in the nitrogen-fixing zone [109]. An *oxyR* mutant in *A. caulinodans* also has an increased sensitivity to H_2_O_2_ [69]. The regulator positively controls the expression of the *katG* gene encoding a bifunctional catalase-hydroperoxidase, involved in detoxification of exogenous H_2_O_2_. Both OxyR and KatG are critical for nodulation and nitrogen fixation with *S. rostrata* [69]. In other rhizobia, *oxyR* is similarly localized in front of the *katG* gene and most likely controls its H_2_O_2_-dependent expression [110].

The LsrB TR of *S. meliloti* is also a LysR-like regulator. An *lsrB* mutant was originally selected for its symbiotic defect during the interaction with *M. sativa*, and induced ineffective, early senescing nodules with abnormal bacteroid differentiation [111]. Thereafter, LsrB inactivation was found to increase strain sensitivity to oxidative stress. This TF positively regulates the expression of genes involved in GSH homeostasis (*gshA*, *gshB*, *gor* gene encoding GR) and the biosynthesis of lipopolysaccharide (*lrp3*-*lpsCDE* operon), which promote both IT formation and bacteroid survival in mature nodules [112]. LsrB was shown to bind to the promoters of *gshA* and *lrp3,* and its activity to be modulated by the redox state of three reactive cysteines [113].

In many bacteria, the regulators of the MarR family control the expression of genes involved in the resistance against various environmental and cellular toxic compounds such as antibiotics, detergents, or ROS [114]. Members of the MarR/OhrR subfamily act as dimeric repressors, which are inactivated by thiol-oxidation and dissociate from the promoter DNA of target genes. They respond to organic hydroperoxides and control the expression of peroxidase-encoding genes, often located in the immediate vicinity of the OhrR regulator. In *S. meliloti* and *A. caulinodans*, OhrR was shown to regulate *ohr* by direct binding to its promoter region and OhrR oxidation to prevent this binding and promote *ohr* expression [70,100]. During S. *meliloti*/*M.sativa* interaction, *ohr* transcripts were detected in nodules and *ohr* inactivation affects the efficiency of *A. caulinodans/* *S. rostrata* symbiosis, suggesting that the sensing of organic peroxides by OhrR plays a role in the adaptation to the host environment [70,115].

In addition to redox-sensing TR, alternative sigma factors of the RNA polymerase may also contribute to the oxidative stress response. Alternative sigma factors enable bacteria to drive the transcription of genes of shared function in response to environmental stimuli. They are particularly abundant in bacteria experiencing a variety of lifestyles, such as rhizobia and endophytic bacteria. For example, the *S. meliloti* genome encodes 14 alternative sigma factors, among which RpoHI is required for nitrogen fixation [107,116]. RpoHI controls gene expression in response to pH stress, heat shock, and stationary phase. The RpoHI regulon was defined by transcriptomic studies in free-living bacteria exposed to heat shock or low pH; it includes various genes of the antioxidant defense (*gshA*, *gshB*, *gor*, *grx1*, *trxA*, *trxB*, *msrA1*, *msrA2*, *msrA3*, *msrB1*, *msrB2*) [117,118]. Moreover, RpoHI was recently shown to play a role in maintaining the cellular redox status [101]. In *R. etli*, RpoH1 is mainly involved in heat-shock and oxidative responses. An *rpoHI* mutant has an increased sensitivity to various oxidants in free-living conditions and forms early senescing nodules with bean [119]. Some other sigma factors that belong to the extracytoplasmic function (ECF) family were also involved in response to various stress conditions, including oxidative stress [53,58,120]. In particular, RpoE4 in *R. etli*, which contributes to tolerance against osmotic and oxidative stresses, controls the expression of the catalase gene *katG*; and RpoE2, a major global regulator of the general stress response in *S. meliloti*, controls the expression of the catalase gene *katC* [121,122,123].

Several data also concern the transcriptional response to oxidative stress in the endophytic bacterium *A. brasilense*. Among the ten alternative RpoE sigma factors that *A. brasilense* possesses, RpoE1 and RpoE2 are activated by photooxidative or oxidative stress through dissociation from their cognate anti-sigma [124]. This leads to indirect induction of the carotenoid biosynthetic pathway via a subset of alternative RpoH sigma factors [58,120,125]. In addition, the regulation of a few genes involved in ROS scavenging has been analyzed. The *ahpC* gene is located on the chromosome, adjacent to an *oxyR*-like gene (*oxyR1*), and its promoter region displays a putative RpoE2-dependent motif [55,124]. The expression of *ahpC* is negatively regulated by OxyR1 under reducing conditions and is positively regulated by RpoE2 [126]. Another *oxyR* copy (*oxyR2*), located adjacent to the *katAII* gene and in opposite orientation, is necessary for the H_2_O_2_ induction of *katAII* gene expression [58].

## 6. ROS Transcriptional and Post-Translational Control of Nodule Metabolism during Nitrogen Fixation

BNF is a highly energy-demanding process whereby 16 ATP are hydrolyzed per mol of reduced N_2_ [127]. However, the Mo-Fe-S complex nitrogenase enzyme is irreversibly inactivated by O_2_. The high aerobic respiratory turnover, in particular, the one maintained by the plant carbohydrates in bacteroids to sustain nitrogenase activity, as well as respiratory protection used by free-living diazotroph to decrease the internal oxygen concentration, are assumed to generate ROS as a side product. Accordingly, the nitrogenase is inactivated by a subtoxic increase of ROS level, as observed for the sugarcane isolate *Gluconobacter diazotrophicus* [61]. In addition to a central regulation exerted by O_2_ level, the nitrogen-fixing bacteria have evolved ROS-dependent regulation of nitrogen fixation metabolism. In endophytic diazotrophs, the genes involved in the synthesis and function of nitrogenase (*nif* genes) are coordinately regulated via the NifLA proteins [128]. NifL is a flavoprotein that modulates the activity of the TR NifA by direct protein-protein binding in response to changes in O_2_ and N_2_ concentrations, the ATP/ADP ratio, and the redox status of the cells [129,130]. In *K. pneumoniae*, the reduced quinone pool generated by the respiratory chain has been proposed to reduce the FAD domain of NifL under anaerobic conditions, leading to the release of NifA and subsequent induction of *nif* genes [131]. In diazotrophic bacteria that lack NifL, like rhizobia or free-living bacteria such as *Herbaspirillum*, the O_2_ regulation of NifA activity involves an invariant motif Cys-X4-Cys that have been proposed to be redox-sensitive [60,132]. In rhizobia, the FixLJ-FixK cascade positively regulates the *nif* genes together with genes involved in adaptation to microoxic conditions. The FixL protein contains a heme moiety, which can bind oxygen, thus inactivating the protein [128]. A correlation between the negative redox potential shift provoked by O_2_ binding to heme and FixL inactivation has been emphasized in *R. etli* [133]. In *B. japonicum*, the TR FixK2 amplifies the response under a moderate decrease of oxygen level to approximately 200 target genes. Its activity depends on a redox control, involving a critical cysteine residue near the DNA-binding motif whose oxidation results in FixK2 inactivation [134]. In parallel, a second hierarchical cascade RegSR-*nifA*, controls the expression of *nif* genes irrespectively of the oxygen conditions [135]. RegSR is a redox-sensitive two-component regulator system, where the sensor component RegS contains a conserved quinone binding site and a conserved redox-active cysteine, suggesting that the membrane-localized quinone pool and/or the active cysteine mediate the redox signal [134,136].

The post-translational regulation of protein activity has been highlighted for various proteins of nitrogen-fixing bacteroids. Among the 20 sulfenylated proteins identified in *S. meliloti* bacteroids, the NifK and NifH components of the nitrogenase were susceptible to sulfenylation [137]. In addition, a large proportion of the sulfenylated protein is involved in the TCA cycle allowing a coordinated regulation between the cellular redox state, the nitrogenase activity, and the enzymes of cellular energy metabolism. A fine control of energy demand and reducing power produced via the TCA cycle might involve polyhydroxybutyrate (PHB), a polyhydroxyalkanoate polymer that acts as carbon storage compounds, and regulates the cellular NAD(P)H pool, thereby being involved in cellular redox balance [138]. The PHB synthesis depends on a three-step pathway involving successively the β-ketothiolase PhbA, the acetoacetylcoA reductase PhbB, and the PHB synthase PhbC, while PhaZ initiates the depolymerization of PHB. Genes involved in PHB synthesis have been characterized in different endophytic bacteria and rhizobia, such as *A. brasilense*, *H. seropedicae*, *S. meliloti*, *R. etli, R. leguminosarum, B. japonicum,* or *A. caulinodans* [82,83,84,139,140,141,142]. The level of ROS was approximately 50% higher in a *phbC* mutant of *H. seropedicae*. This feature leads to the inactivation of the FixK homolog, FnR, a [Fe-S] containing protein that should be maintained in its reduced form to induced genes involved in the adaptation to microaerophilia [140]. The *H. seropedicae phbC* mutant fails to promote plant growth, although it conserves the ability to colonize plant roots as the wild type [63]. The PHB synthase is also not essential for the colonization of *A. brasilense* [143]. In *A. caulinodans*, a *phbC* mutant was deprived of nitrogenase activity, depleted in ATP and displayed an increase in reduced NADH [83]. Moreover, the *nifA* expression, controlled by the FixLJK cascade, was totally abolished, and nodules induced by the PHB mutant were devoid of bacteroids (Table 1). Similarly, the *S. meliloti phbC* mutation results in the formation of numerous nodules that are unable to fix nitrogen [82]. However, even if the PHB accumulation in bacteroids occurs in soybean and common bean nodules, and not in pea, alfalfa, and clover, [144], the *phbC* mutation of *B. japonicum, R. leguminosarum*, and *R. etli* does not impair nitrogen fixation [84,141]. Terpolilli and colleagues proposed that bacteroids used multiple lipid sinks, as fatty acid or glycolipids, in addition to PHB, for excess reductants and regulation of the redox balance in fixing symbiosis [145]. Thus, the importance of the PHB cycle depends on the physiology of the nodule.

## 7. Conclusions

During the last years, the characterization of many redox regulatory systems has shown the importance of redox regulation in the plant-bacteria symbiotic recognition and the functioning of the symbiotic interactions. The specific redox regulation in the symbiotic interaction may have a crucial role in differentiating pathogens from symbiotic microorganisms and to allowing the proper functioning of the interaction. The development of omics data analyses gives us a more general view of the redox network regulation and the expression of different members depending on the physiological state of the organisms. However, these advances are revealing the complexity of the regulatory mechanisms and an increased number of key regulatory actors depending on the stage of the analysis and the biological material. Many lines of research remain to be opened. One of them is to compare the different redox regulation occurring in the different symbiotic interactions to identify similar and different processes. These analyses may allow defining a regulatory hub used by partners in different types of symbiotic relations. Another crucial question is how the organisms regulate their responses in front of multiple stimuli, including biotic and abiotic environments and the metabolic processes. Finally, the screening of potential candidates involved in improving the plant-microbe symbiotic interactions to increase the efficiency of plant development and defense is of high interest to develop more sustainable agriculture.

## Figures and Tables

**Table 1:** Genes of diazotrophic rhizobia involved in cellular redox homeostasis and in the efficiency of endophytic/endosymbiotic interaction. Phenotypes *in planta* are the results of gene inactivation except for *S. meliloti katB* mutant that overexpresses *katB*. PHB, polyhydroxybutyrate.

Genes	Bacterium	Mutant Phenotype *In planta*	References
	**ENDOPHYTES**		
*sod*	*G. diazotrophicus*	impaired root colonization	[62]
*gr*	*G. diazotrophicus*	impaired root colonization	[62]
*phbC*	*H. seropedicae*	loss of plant growth promoting ability	[63]
	**ENDOSYMBIONTS**		
**ROS scavenging**			
*sodA*	*S. meliloti*	depending on the *S. meliloti* strain genotype	[64]
*katB*	*S. meliloti*	delayed nodulation; aberrant and enlarged infection threads	[28]
*katB/katC*	*S. meliloti*	altered bacteroid differentiation	[65]
*katA/katC*	*S. meliloti*	early nodule senescence	[65]
*katE*	*M. loti*	lower nitrogen fixation capacity	[66]
*katG/prxS*	*R. etli*	lower nitrogen fixation capacity	[67]
*prxA*	*M. huakii*	lower nitrogen fixation capacity	[68]
*katG*	*A. caulinodans*	decreased number and nitrogenase activity of stem nodules	[69]
*ahpCD*	*A. caulinodans*	decreased number and nitrogenase activity of stem nodules	[70]
*ohr*	*A. caulinodans*	decreased number and nitrogenase activity of stem nodules	[71]
**GSH/Grx system**			
*gshA*	*R. leguminosarum*	impaired root colonization, lower nitrogen fixation capacity	[72]
*gshB*	*R. leguminosarum*	impaired root colonization, lower nitrogen fixation capacity	[72,73]
*gshB*	*S. meliloti*	delayed nodulation, early nodule senescence	[74,75]
*gshB*	*R. etli*	delayed nodulation, early nodule senescence	[76]
*gshB*	*R. tropici*	low competitiveness for nodule occupancy, early nodule senescence	[77]
*gshB*	*M. huakii*	early nodule senescence	[78]
*gor*	*S. meliloti*	low competitiveness for nodule occupancy, lower nitrogen fixation capacity	[79]
*grx* (*RL2615*)	*R. leguminosarum*	impaired root colonization	[72]
*grx1*	*S. meliloti*	altered bacteroid differentiation	[80]
*grx2*	*S. meliloti*	early nodule senescence	[80]
*grx1/grx2*	*A. caulinodans*	lower nitrogen fixation capacity	[81]
*grx3/grx4*	*A. caulinodans*	lower nitrogen fixation capacity	[81]
**PHB metabolism**			
*phbC*	*S. meliloti*	lower nitrogen fixation capacity	[82]
*phbC*	*A. caulinodans*	loss of nitrogen fixation ability	[83]
*phbC*	*B. japonicum*	low competitiveness for root colonization	[84]
